# Recurrent intestinal ulcer with bloody stool for >10 years in an adolescent boy of 15 years: A case report

**DOI:** 10.1097/MD.0000000000034096

**Published:** 2023-06-23

**Authors:** Wei Li, Yang Wang, Yufang Wang, Chuan Yang, Zhen Lu, Zhaoxia He, Lan Peng

**Affiliations:** a Wenjiang District People’s Hospital, Chengdu, China; b West China Hospital of Sichuan University, Chengdu, China.

**Keywords:** adolescent, Chron’s disease, intestinal ulcer, nutritional support

## Abstract

Infectious diseases, autoimmune diseases and vascular diseases can lead to intestinal ulcers, and inflammatory bowel disease is typically suspected as the underlying cause of ulcer and bleeding from the intestinal ulcers leading to bloody stool, and malnutrition. Here we report a rare case of successfully treated case of intestinal ulcer, bloody stool, and malnutrition by dietary modification and avoidance of long term barbecued spicy food and carbonated drinks.

**Patient concerns:**

A 15-year-old male patient had repeated symptoms of blood in the stool for >10 years. Treatment for Chron’s disease was not successful. An exhaustive investigation failed to confirm the diagnosis.

**Diagnosis interventions:**

Through changing diet structure, avoiding spicy food, and supplementing enteral nutrition and recurrent glutamine.

**Outcomes:**

The patient’s symptoms improved significantly, and the intestinal ulcer healed under endoscope.

**Lessons:**

Pay attention to healthy diet in life and avoid long-term consumption of spicy food and carbonated drinks.

## 1. Introduction

Intestinal ulcers can have various symptoms, such as abdominal pain, diarrhea, and blood in the stool. Ulcerative colitis, Crohn’s disease (CD), infectious enteritis, and ischemic enteritis can all cause intestinal ulcers. The detection rate of intestinal ulcers has increased significantly with the popularity of endoscopy.

Dietary fiber has complex properties, including nutritional effects on the intestinal mucosa, production of volatile fatty acids, changes in bacterial flora, and fecal bile acids. Gastrointestinal diseases are usually caused by the lack of specific nutrients.^[1]^

The inflammatory bowel disease (IBD) Professional Committee of the China Medical Education Association points out that IBD has become one of the most common and difficult digestive diseases in China, requiring more input in basic research, clinical diagnosis, and treatment.^[2]^ The IBD mostly starts in adolescents and is considered a recurrent, progressive, and disabling disease with serious effects on normal life.

We report a malnourished adolescent male patient of 15 years who had >10 years of history of repeated abdominal pain, blood in the stool, and anemia. He had visited several hospitals. Colonoscopy showed intestinal ulcers. He was managed for suspected CD but there was no improvement. Further exhaustive investigations did not confirm Chron’s. After carefully reviewing his medical history, we discovered that he had an unhealthy food habit of drinking carbonated beverages and spicy barbecued food. Finally, after 2 months of correcting dietary habits and lifestyle, together with enteral nutrition support his symptoms resolved, and follow-up colonoscopy showed healing of ulcers.

Written informed consent was obtained from the patient’s legal guardian for the publication of the case report.

## 2. Case report

A 15-year-old male was admitted for blood in the stool for 2 days and fainting attacks.

He had a history of bloody stool on and off for >10 years, accompanied by abdominal pain, anorexia, fatigue, and a weight loss of 15 kg. He had visited many hospitals. According to the family members, CD was suspected. The symptoms persisted and were aggravated after spicy foods. There was no fever, night sweats, joint pain, and skin lesions. The enhanced CT scan of the abdomen revealed scattered gas in the colon, segmental swelling and thickening of the colon and rectum, enlarged mesenteric and retroperitoneal lymph nodes, enlarged spleen, and a small amount of pelvic fluid. Colonoscopy was suspicious of CD. Sigmoid colon biopsy showed severe chronic mucosal inflammation, ulceration, crypt abscess, and lymphoid tissue hyperplasia in the lamina propria. Gastroscopy revealed chronic non-atrophic gastritis and duodenitis. Capsule endoscopy showed chronic superficial gastritis, inflammation of the descending duodenum, unspecific enteritis, and diffuse colonic inflammation. He was managed for Crohn’s, but the intermittent bloody stool and abdominal pain persisted.

Two days before his current hospital admission, he was treated in a local hospital for bloody stool and fainting attack. The reports from the local hospital revealed hemoglobin of 3.1 g/dL. He had a blood transfusion of 3.5 units and was transferred to our hospital for further management.

**Figure 1.**
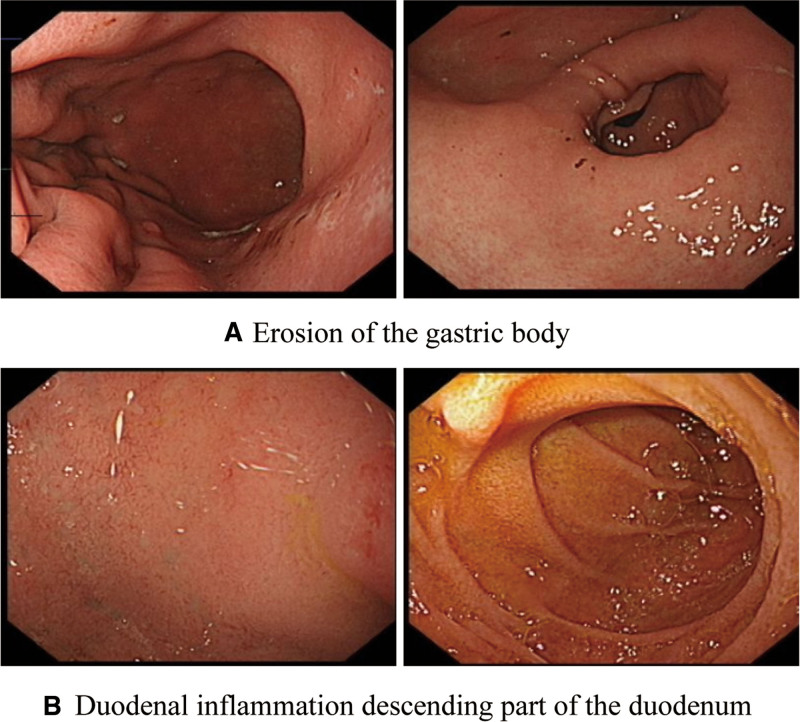
Gastroscope findings. (A) Erosion of gastric body. (B) Duodenal inflammation descending part of the duodenum.

**Figure 2.**
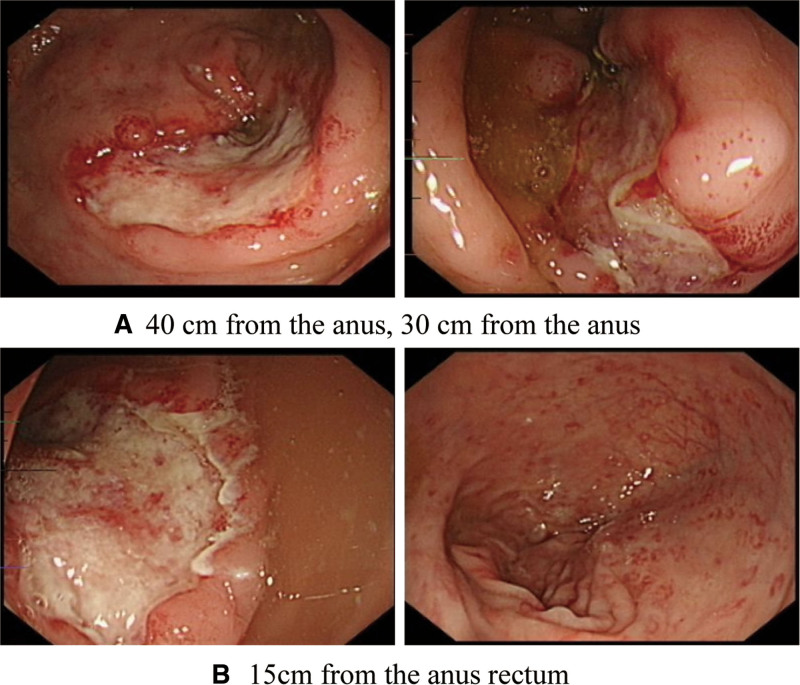
Colonoscopy findings: (A) 40 cm from the anus, 30 cm from the anus and (B) 15 cm from the anus.

On admission, his vitals were stable. He was anemic and malnourished, with a BMI of 16. The abdomen was soft and non-tender. The C-reactive protein was 85.3 mg/L. The reticulocyte count, immunoglobulin, tuberculosis antibody, and erythrocyte sedimentation rate were normal. Anti-nRNP antibody was positive, antineutrophil cytoplasmic antibodies was negative, anti-Saccharomyces Cerevisiae antibodies: IgA was significantly increased, and T-SPOT TB was negative. Gastroscopy revealed chronic non-atrophic gastritis with erosion, bile reflux, and duodenitis (Fig. 1). Gastroscopy biopsy revealed acute on chronic inflammation of superficial gastric mucosa, and negative immuno-histochemistry findings. The biopsy from duodenal bulb revealed chronic active inflammation of the superficial mucosa. On colonoscopy (scoped up to 40 cm only due to poor bowel preparation) there were large, deep concave ulcers at 40 cm, 30 cm, and 15 cm from the anus. There was a white coating of normal mucosa between the ulcers. Scattered, congested and erosive foci were seen in the rectal mucosa (Fig. 2). Colon biopsy at 30 cm revealed superficial mucosa with loose and edematous lamina propria, and infiltration of few lymphocytes, plasma cells, and neutrophils. Pathological biopsy of the rectum showed edema in the lamina propria of the whole mucosa with infiltration of lymphocytes, plasma cells and neutrophils; Accompanied by cryptitis, crypt deformation, branches, and crypt atrophy, but no clear crypt abscess. There was no granuloma seen in the mucosal tissues submitted for histopathology examinations.

The CT enterography revealed thickened gastric wall in the antrum, and segmental thickening of the transverse, descending and, sigmoid colon with localized luminal narrowing. The CT also revealed enlarged spleen.

Patient was treated with mesalazine enteric-coated tablets, compound Glutamine Entersoluble capsules, iron sucrose infusion, red blood cell transfusion, enteral nutrition powder (Ellendo), and other symptomatic treatment. His conditions improved, and there was no abdominal pain, diarrhea, or bloody stool.

**Figure 3.**
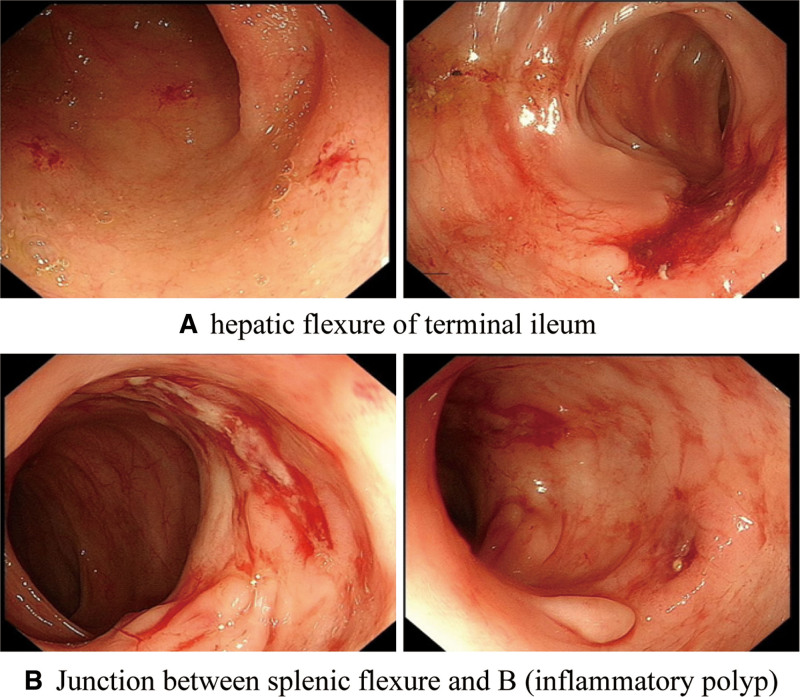
Colonoscopy findings: (A) hepatic flexure of the terminal ileum and (B) the junction between splenic flexure and B (inflammatory polyp).

A repeat colonoscopy (Fig. 3) showed healing ulcers in the terminal ileum and colon.

## 3. Discussion and conclusions

This patient, a 15 years male, malnourished had a history of bloody stool for >10 years. He was treated several hospitals for suspected CD without significant improvement. Finally he responded well and improved with enteral nutrition and diet modification.

The nonspecific intestinal ulceration (chronic colitis) in this patient may be due to physical and chemical stimulation of the gut by spicy food and carbonated drinks. Malnutrition is known for decreased response to drug therapy and increased risk of sepsis and mortality.^[3]^ Enteral nutrition is the choice of treatment for CD.^[4]^ The CD is a chronic, nonspecific, relapsing, inflammatory bowel disease. It usually starts in adolescents and affects almost all organs and systems. Colonoscopy has characteristic findings of segmental longitudinal ulcers, and noncaseating granulomas on histopathology.^[5]^

The proportion of chronic colitis is relatively high in preschool and school ages.^[6]^ In children, blood in the stool may be due to colorectal polyps, Meckel’s diverticulum, inflammatory bowel disease, intussusception, necrotizing enterocolitis, etc.^[5]^

In the present case, the antineutrophil cytoplasmic antibodies were negative, and endoscopy and pathology reports did not support CD. Considering his age, he was investigated for Epstein–Barr virus-related T Cellular lymphoproliferative disease. The comprehensive Epstein–Barr virus DNA quantitative examination and repeat biopsy after treatment did not support lymphoproliferative disease.

In this patient, the colitis symptoms improved, and ulcers in the colon healed after enteral nutrition support (Ellendo), together with the introduction of compound glutamine to enhance the repair of the intestinal mucosa and immune function. The hemoglobin level improved and was stable, and there was no abdominal pain or blood in the stool. Repeat colonoscopy showed healing of ulcers.

Long-term physical and chemical stimulation from a spicy diet can lead to nonspecific intestinal ulcers.^[7]^ Repairing the intestinal mucosa, strengthening enteral nutrition, and improving dietary structure can help promote the healing of the intestinal mucosa, improve the natural course of the disease, and relieve intestinal ulcers.^[1]^

In conclusion, a 15-year-old male patient with bloody stool for >10 years and malnutrition was managed for suspected Chron’s disease in several hospital, but his symptoms did not improve. He had >10 years of eating spicy food and carbonated drinks. Exhaustive investigations failed to diagnose the exact cause. Finally, the patient had a successful recovery following enteral nutrition support and the use of compound glutamine, together with a modification of diet to avoid spicy food and carbonated drinks. Limitation of this case report could be difficulty in proving the cause and effect of spicy food and carbonated drinks for the intestinal ulcers leading to blood in stool and malnutrition. However there was significant improvement following modification of dietary pattern by avoiding long term exposure to spicy food and carbonated drinks that let to improvements in clinical scenario of patient. Further study, possibly with more such cases may necessary to further add to our observation.

## Acknowledgments

We thank *Medjaden* Inc. for the scientific editing of this manuscript.

## Author contributions

**Data curation:** Yufang Wang.

**Formal analysis:** Zhen Lu.

**Funding acquisition:** Lan Peng.

**Project administration:** Chuan Yang.

**Software:** Zhaoxia He.

**Writing – original draft:** Wei Li, Yang Wang.

**Writing – review & editing:** Wei Li, Yufang Wang, Chuan Yang, Zhen Lu, Zhaoxia He, Lan Peng.
